# Taming the Sentinels: Microbiome-Derived Metabolites and Polarization of T Cells

**DOI:** 10.3390/ijms21207740

**Published:** 2020-10-19

**Authors:** Lukasz Wojciech, Kevin S. W. Tan, Nicholas R. J. Gascoigne

**Affiliations:** 1 Immunology Programme and Department of Microbiology and Immunology, Yong Loo Lin School of Medicine, National University of Singapore, 5 Science Drive 2, Singapore 117545, Singapore; micluka@nus.edu.sg; 2 Laboratory of Molecular and Cellular Parasitology, Healthy Longevity Programme and Department of Microbiology and Immunology, Yong Loo Lin School of Medicine, National University of Singapore, 5 Science Drive 2, Singapore 117545, Singapore; mictank@nus.edu.sg

**Keywords:** microbiome, metabolites, metabolome, T cells, T regulatory cells, Th17 helper cells, Th lineage polarization, intraepithelial lymphocytes, inflammatory bowel disease, IBD

## Abstract

A global increase in the prevalence of metabolic syndromes and digestive tract disorders, like food allergy or inflammatory bowel disease (IBD), has become a severe problem in the modern world. Recent decades have brought a growing body of evidence that links the gut microbiome’s complexity with host physiology. Hence, understanding the mechanistic aspects underlying the synergy between the host and its associated gut microbiome are among the most crucial questions. The functionally diversified adaptive immune system plays a central role in maintaining gut and systemic immune homeostasis. The character of the reciprocal interactions between immune components and host-dwelling microbes or microbial consortia determines the outcome of the organisms’ coexistence within the holobiont structure. It has become apparent that metabolic by-products of the microbiome constitute crucial multimodal transmitters within the host–microbiome interactome and, as such, contribute to immune homeostasis by fine-tuning of the adaptive arm of immune system. In this review, we will present recent insights and discoveries regarding the broad landscape of microbiome-derived metabolites, highlighting the role of these small compounds in the context of the balance between pro- and anti-inflammatory mechanisms orchestrated by the host T cell compartment.

## 1. Introduction

The innate and adaptive immune systems make up a cooperative set of machinery involved in organismal defense from pathogens and malignant cells, while limiting reactions to self molecules. T cells are a dominant faction of the adaptive arm, for which initial functional diversification occurs in a central organ, the thymus. To some extent, thymic-derived regulatory T cells (tTregs) can maintain immune homeostasis [1,2]. However, it is widely accepted that to fully perpetuate this pro- and anti-inflammatory balance, immune compartments require specialized executors that are reprogrammed in the peripheral organs. T cell receptor (TCR) specificity can play a vital role in those lineages’ polarization [3,4,5]. However, experiments carried out on mouse models with transgenic T cell receptors and nextgen sequence (NGS) data from T cell repertoires has revealed that, in some circumstances, clones that express a particular TCR exhibit divergent capability in terms of functional specialization in peripheral organs [6,7]. This promiscuity of the TCR in the context of different T cell lineages indicates a huge plasticity within the adaptive compartment and a complex network of interacting components involved in building up niches for specific cell subsets.

The intestines constitute a unique, very dynamic, environment that harbors complex microorganism consortia composed of prokaryotes, eukaryotes, and viruses. It is estimated that a healthy human microbiome consists of 500 to 1000 different species belonging to more than 70 genera [8,9]. From the immune compartment’s perspective, gut tissues maintain the most functionally and phenotypically diversified T cell collection. The coexistence of these two complex systems, the microbiota and immune compartment, necessitates a sophisticated network of interacting elements to continuously maintain a beneficial synergy rather than an antagonistic host–pathogen relationship. There is a growing body of evidence that gut dwelling consortia constitute key orchestrators for many aspects of host physiology, including metabolic capacity, host defense and behavioral processes. The quantitative and qualitative impoverishment of the T cell compartment in germ-free animals indicates the great importance of commensal microorganisms during development and peripheral shaping of this cell subset [10,11].

Representatives of the gut microbiome that exert potentially pro-inflammatory [3] and anti-inflammatory [12,13,14] effects on the host immune system have recently been identified [15]. A dysregulated immune response that results in intestinal inflammation is associated with an altered intestinal microbiota, reflected by a decreased diversity of gut-dwelling communities; the phenomenon is called dysbiosis [16]. Interestingly, some of the hallmarks of inflammation are transmittable upon gut microbiome transfer [17], which indicates a pro- or anti-inflammatory imprint associated with the distribution or composition of the species within gut consortia [16,18].

**Figure 1:**
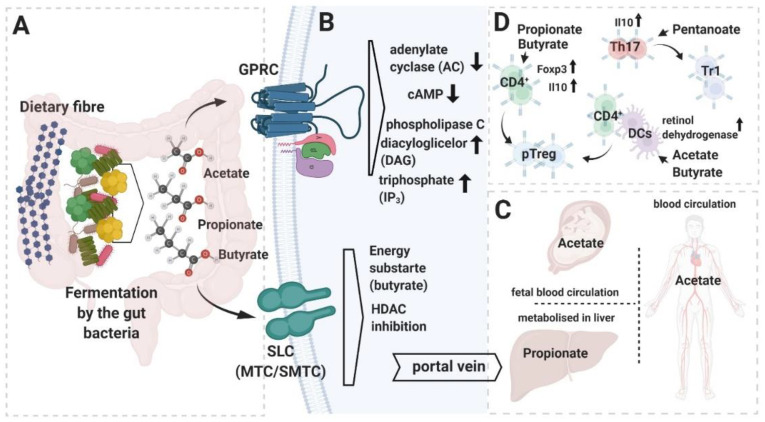
Summary of short chain fatty acids (SCFAs) metabolism and functions. (**A**) Most of the SCFAs are produced in the large intestine upon fermentation of dietary fiber, which is carried out by the host microbiome’s bacterial members. (**B**) SCFAs can activate cell-intrinsic cascades through the g-protein-coupled receptors (GPCRs) (upper panel) or affect transcription of the genes upon inhibition of histone deacetylases HDAC (lower panel). In the gut, SCFAs, after transport into the colonocytes’ intracellular compartment (mostly through monocarboxylate transporter channels (MTC) and sodium-coupled monocarboxylate transporters (SMCT)), are utilized as an energy substrate (butyrate) or channeled further to the blood circulation through the portal vein. (**C**) Systemic distribution of SCFAs. (**D**) Impact of the different SCFAs on T cell lineage polarization. Graphic created with BioRender.

Physiological processes maintained by the microbiome constitute a source of metabolic byproducts (small chemical compounds that make up a uniquely configured signaling network) [18,19]. Besides being essential in ecological interactions between microbiota species, these metabolites serve as crucial signaling molecules engaged in reciprocal host–microbiome interplay [16,18]. It has become apparent that metabolites produced by the microbiota affect many physiological processes, including control of the adaptive arm of the immune system (Figure 1).

In this work, we present an overview of the most prominent metabolites associated with human and rodent models of the gut microbiota. We aim to provide the most recent information regarding the molecular mechanisms involved in the interactions between the host adaptive immune compartment and the microbiome. As these are orchestrated by metabolites, we highlight the role of these small compounds in health and disease.

## 2. Short Chain Fatty Acids (SCFAs)

The composition of the diet appears to be a critical factor determining human health and wellbeing. Besides broadly defined “nutrients” produced by the host digestive enzymatic machinery, digestion of a large fraction of the diet relies upon gut-residing microbes. Dietary fibers and plant-derived carbohydrates represent abundant components of everyday food [19]. According to its physical properties, fiber is grouped into soluble, insoluble, or resistant starch [20]. These latter polymers exhibit extraordinary resistance to the entire landscape of host-related enzymes in the digestive system. The breakdown of the dietary fiber occurs primarily in the colon, and this process is coordinated by microbiome-derived carbohydrate-active enzymes (CAZymes) [21]. Fermentation of fiber in this anaerobic environment constitutes an important carbon source for commensal anaerobic bacteria and host cells in the large intestine milieu [22,23]. The major end products of this process are the short-chain fatty acids (SCFAs) that quantitatively constitute the most abundant bacterial-derived metabolite in the gut environment [19]. Acetate, propionate, and butyrate, along with other SCFAs, provide approximately 10% of total absorbed calories [24]. Importantly, the molar distribution of individual SCFAs in the entire pool of bacteria-produced metabolites depends upon diet and the composition of the gut microbiome. Different preferences in terms of SCFA production by some gut-associated bacterial strains are summarized in Table 1. The concentration of SCFAs reaches its maximum in the distal cecum and proximal colon, decreasing towards the distal colon [19]. Basolaterally and apically expressed H^+^- and Na^+^-coupled transporters, respectively monocarboxylate transporters (MCT)1 (*SLC16A1*), MCT4 (*SLC16A3*), sodium-coupled MCT (SMCT)1 (*SLC5A8*), and SMCT2 (*SLC5A12*), account for cellular uptake of SCFA, being the major route for entry for these compounds into the colonocytes’ intracellular compartments [25]. Butyrate is utilized locally by the colonocytes as a favored energy source [19]. The other absorbed SCFAs are channeled through the portal vein, and can be metabolized in the liver (propionate) or released further into the blood circulation (acetate) [19]. The effects of butyrate are mostly assigned to the gut compartment, where this compound reaches a biologically effective concentration. At the same time, acetate, because of its systemic availability, exhibits more multiorgan activity in regard to the host’s physiological processes.

Besides their undeniable nutritional value, SCFAs serve as important multimodal signaling molecules within the host–microbiome interactome (Figure 1). Among microbial-produced SCFAs, butyrate, pentanoate, and to a lesser extent, propionate, exhibit histone deacetylase (HDAC) inhibitory activity [29]. Thus, by affecting chromatin structure, these compounds regulate the expression of numerous genes with heterogeneous functions [27,29,30,31,32,33]. Genes upregulated upon butyrate-associated HDAC inhibition include those related to the apoptosis cascade (cyclin dependent kinase inhibitor 1A (CDKN1A), GATA2, protein kinase C delta (PKCD)), cell differentiation (BAK, interleukin 8 (IL8)), and cell-cycle arrest (retinoic acid receptor beta (RARβ), transglutaminase TG1, cyclin E, carboxypeptidase A3 (CPA3), CD86, intercellular adhesion molecule 1 (ICAM1)) [29].

Another mode of SCFA interaction with the intracellular machinery is through the activation of mammalian G protein-coupled receptors (GPCR) [34,35]. The entire GPCR cluster comprises around 800 different cell surface receptors, and to date, three of them have been classified as mediators of biological effects triggered by SCFAs [36]. Free fatty acid receptor 3 (FFAR3 or GPR41), FFAR2 (GPR43), and GPR109A show different tissue distribution and ligand specificity. GPR41 is expressed on enteric neurons and intestinal endocrine cells (together with GPR43), while GPR43 is found on white adipose tissue, gut epithelium, innate immune cells, and gut Tregs. The expression of GPR109a is restricted to dendritic cells (DC), adipose tissue, and keratinocytes. Among all SCFA-activated receptors, GPR41 and GPR43 exhibit more promiscuous preferences in terms of ligand binding and, as such, can be activated by acetate, propionate, and butyrate [37]. Butyrate or β-hydroxybutyrate, a butyrate breakdown product of ketogenesis, constitute favorable ligands for GPR109A activation. 

The Gi/o proteins, common partners for all three SCFA-activated GPCRs, are considered to be inhibitory for adenylate cyclase (AC) and potassium channels. Thus, downstream signal transduction after SCFA-associated GPCR activation leads to diminished activity of AC, which in consequence inhibits cyclic adenosine monophosphate (cAMP) synthesis [38,39]. Additionally, a component of the GPR43 signalosome (a G_q/11_ protein) stimulates phospholipase C (PKC), which in turns leads to diacylglycerol (DAG) and inositol trisphosphate (IP_3_) production, resulting in the activation of PKC and calcium signaling [40]. The β-arrestins constitute a key regulator of GPCR function, contributing positively in extracellular signal-regulated protein kinase (ERK)1/2, mitogen-activated protein (MAP) kinase phosphorylation. Strong evidence indicates the involvement of the β-arrestins in the intracellular transduction of the signals initiated by activation of GPR109A and GPR43 [41]. Furthermore, GPR43 and GPR41 can be expressed together with α-gustducin, a protein that plays a role in the signaling complexes initially found in taste cells [42]. The α-gustducin-dependent glucagon-like peptide 1 (GLP-1) release in colon is probably mediated by these fatty acid receptors [42,43]. Thus, diversification of the partners recruited during signal induction upon GPCR activation and ligand preferences might explain different, tissue-specific or SCFA-specific biological outcomes driven after exposure to these compounds.

## 3. The Role of SCFAs in T Cell Driven Immune Homeostasis

During the last decade, the role of the SCFAs has been heavily investigated in the context of multiple aspects of human health. There is a substantial body of evidence that these bacterially-derived metabolites can modulate the immune system’s adaptive arm. Tregs, as a part of the adaptive immune system, play a central role in immune tolerance. A striking example of how this T cell compartment is essential for maintaining the balance between pro- and anti-inflammatory responses are the phenotypes of human immunodysregulation polyendocrinopathy enteropathy X-linked (IPEX) syndrome [44] and the Scurfy mice model [45]. These two fatal autoimmune disorders are associated with a mutation within the *FOXP3* gene that results in arrested development of the Treg compartment and consequently leads to multiorgan autoimmune reactions. Thus, the composition of microbiome metabolomes and the distribution of SCFAs within the landscape of metabolites likely affects the Treg subset and has a profound impact on overall immune homeostasis. 

Two independent groups identified butyrate as a microbiota-produced factor (fermentation by product of *Clostridiales*) that positively affects Tregs in the gut via epigenetic control of *Foxp3* gene expression [32,33]. These teams showed that the mechanism by which Treg induction is controlled is by HDAC inhibition. Butyrate-driven increased acetylation of histones, particularly H3 within the *Foxp3* gene, induces methylation of the conserved non-coding DNA sequence elements of the *Foxp3* enhancer [46] and, consequently, augments conversion of conventional CD4 T cells towards the Treg phenotype. It is worth noting that the effect of butyrate in these experimental models was restricted to the so called extrathymically differentiated Treg (peripheral or pTreg) and that this particular SCFA had no effect on other CD4 T cell subsets. Propionate (in the altered Schaedler flora, this SCFA is produced by the *Clostridium ramosum* (XVII) to a large extent), similarly to butyrate, exerted a positive impact on the colonic CD4 regulatory compartment in experimental mouse models [27]. This compound specifically enhanced Foxp3^+^ IL10-producing Tregs. However, the mechanism postulated by the authors differs from that orchestrated by butyrate. In the case of this SCFA, the intrinsic cascade of events in Treg cells was induced upon activation of the surface GPR43 receptor.

The modes by which SCFAs affect the Treg compartment are not limited to direct induction of Foxp3 and IL10 expression, either by HDAC inhibition or by sensing GPR receptors, within or on the surface of CD4 T cells, respectively. CD103-expressing DCs represent a mucosal component actively involved in pTreg generation by promoting their differentiation from naive T cells [47]. Tan and colleagues demonstrated that activation of G-protein coupled receptors (GRC43 and 109) on DCs by gut-derived SCFAs enhanced retinol dehydrogenase activity and changed these cells’ competence towards becoming more effective pTreg “generators” [48]. Interestingly, only acetate and butyrate, but not propionate, exhibited this effect on the Treg compartment’s activity. Furthermore, animals fed with a diet enriched with dietary fiber were characterized by an increased immunoglobulin A (IgA) level and a significantly higher proportion of T-follicular helper (Tfh) cells.

By selecting beneficial bacterial communities and reducing colonization of potentially pathogenic species, IgA constitutes an essential mediator of host–commensal symbiosis [49]. The secretion of this immunoglobulin can be controlled by T cell-independent and T cell-dependent mechanisms [50,51]. In the former scenario, Tfh cells contribute to B cell clonal selection and promote class switching within germinal centers (GCs). Importantly recent discoveries revealed innate lymphoid cells (ILCs), in particular ILC3, as a key player in T cell-dependent IgA+ B cell response [51]. Postulated mechanisms for this pertain to regulation of Tfh activity in an MHC class-II dependent manner. Notably, according to the new report, SCFAs were highlighted as a commensal microbiota-produced metabolite that affects the expansion of ILC1, ILC2, and ILC3 in the intestines by activation of their G-protein-coupled receptors [52].

Another SCFA for which potent effects on the CD4 compartment were recently identified is pentanoate [53]. Similar to butyrate, pentanoate exerted HDAC inhibitory activity, but in contrast, pentanoate had no effect on Tregs. Upon pentanoate exposure, CD4 effectors (predominantly Th17) were affected and functionally skewed towards IL10-producing cells. Moreover, intrinsic effects of SCFA are not restricted to epigenetic modification. Expression of the anti-inflammatory cytokine IL10 in the Th17 compartment was upregulated by the pentanoate-induced metabolic shift, due to signaling through the mTOR/AKT axis.

Type one diabetes (T1D) is an autoimmune disease mediated by T cells. Malfunction of the CD4 compartment in T1D is in part associated with functional imbalance of the Foxp3^+^ Treg subset [54]. While destruction of insulin-producing pancreatic β-cells in this disorder has a genetic provenance, the composition of the intestinal microbiome in diabetic patients and T1D animal models constitutes an important contributor in accelerating or preventing disease progress [55,56,57]. Metagenomic analysis revealed that a substantial decrease in microbiome-derived gene clusters was associated with overall SCFA production [23], and a severely reduced abundance of butyrate producers from the genus *Roseburia* [58] is corelated with T1D and long-standing T1D, respectively. Some insights into the mechanistic aspects attributed to SCFAs related to the development T1D were revealed in non-obese diabetic (NOD) mice [59]. Using a “pro-butyrate” and “pro-acetate” specified diet (butyrylated or acetylated high-amylose maize starch), Mariño and colleagues revealed that in the NOD mice model, both butyrate and acetate exhibit protective effects on T1D development by orchestrating distinct mechanisms [57]. An enlarged Treg compartment was found after exposure to an elevated concentration of butyrate. This was attributed to increased H3K9 histone acetylation and enhanced *Foxp3* transcription. While butyrate-driven, Treg-yielding reprogramming confirmed the previously reported HDAC-related mechanism, an acetate-mediated, impaired generation of diabetogenic CD4^+^ and CD8^+^ T responders was associated with intrinsic remodeling of B cells upon GPR43 activation [57].

Another study revealed new insights linking the microbiome that produces SCFAs and central tolerance development in the prenatal stage [60]. The importance of maternally-derived acetate on fetal T cell progression was demonstrated in the context of preeclampsia, a common pregnancy disorder characterized by decreased Treg numbers [61,62]. Importantly, immune changes related to preeclampsia are attributed to the maternal and fetal immune compartment [60,63] and, as such, this disorder carries a higher risk of cardiovascular disease or allergy in offspring [64,65]. It was found that that circulating maternally-derived acetate has a positive impact on thymic Treg development in the fetus. Interestingly, the mechanism driven by SCFA involved in rescuing tTreg differentiation required modulation of the autoimmune regulator (AIRE) expression, a vital transcription factor expressed in medullary thymic epithelial cells (mTEC) which orchestrates negative selection and tTreg development in response to tissue-specific antigens (e.g., pancreas antigens) [66,67].

Therapies based on checkpoint blocking antibodies to antigens like PD-1 or CTL-4 have raised new hope for patients with advanced cancer [68,69,70]. Recent studies indicate that the composition of the microbiome is one of the factors associated with clinical outcome in patients undergoing treatment with immune checkpoint blockade [71,72]. Work done on French cohorts treated with ipilimumab, a blocking CTL-4 therapeutic, revealed that low baseline expression of butyrate and propionate was associated with longer progression-free survival [73]. Indeed, butyrate reduced the efficacy of CTLA-4 blockade in a mouse model, and the mechanism by which SCFAs restrained ipilimumab effectiveness involved the modulation of B7 (CD80 and CD86) expression on the surface of DCs and inducible T-cell costimulator (ICOS) within T cells.

## 4. Tryptophan Metabolites

### 4.1. Synthesis and Biological Activity

**Figure 2:**
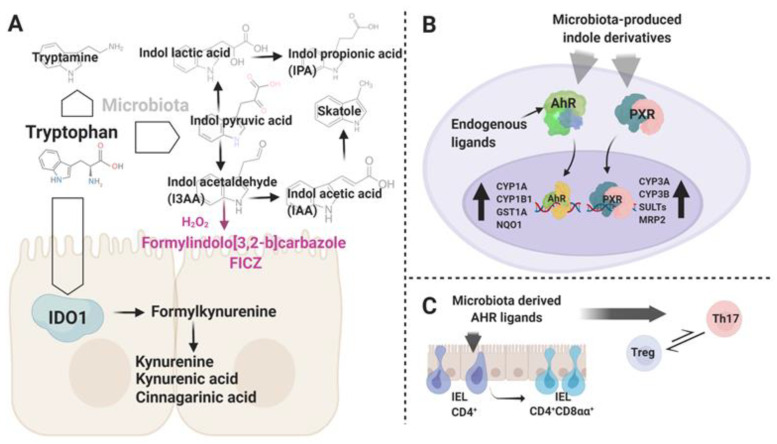
(**A**) Schematic representation of the endogenous and microbiota-associated pathways engaged in tryptophan breakdown in the gut environment. Highlighted in violet is a putative light-independent synthesis pathway of 6-formylindolo [3,2-b]carbazole (FICZ), an endogenous aryl hydrocarbon receptor (AHR) ligand. (**B**) The Trp metabolites act as AHR and pregnane X receptor (PXR) ligands. Upon activation, AHR and PXR translocate into the nucleus and activate the transcription of target genes. (**C**) AHR stimuli play an important role in the development of intraepithelial lymphocytes (IEL) subsets and in control of the Treg–Th17 axis. Graphic created with BioRender.

Amino acid metabolites belong to another chemically diverse group of compounds that impact host health by a vast spectrum of mechanisms [74]. Malfunctions of the immune system and altered composition of the gut microbiome constitute hallmarks of mice fed with a tryptophan-depleted diet [75]. These observations indicate that tryptophan (Trp) and its derivatives are crucial orchestrators of intestinal immunity and ecological ties within gut microbial communities (Figure 2). The kynurenine pathway (KP) and, to a minor extent, the serotonin pathway, are both involved in the endogenous breakdown of ingested Trp to metabolites like kynurenine, kynurenic acid (plus many others), and serotonin, respectively. [76]. Besides being endogenously processed, a substantial fraction of diet-derived Trp is metabolized by a broad representation of gut-dwelling microbiota. For some lactic acid bacteria, in particular environmental conditions, tryptophan can be an alternative to sugars as an energy source. Thus, this amino acid’s catabolism might constitute an evolutionary fit that gives bacteria a metabolic advantage in the highly complex and competitive intestinal ecosystem. More than a hundred years ago, *Escherichia coli* and *Vibrio cholerae* were identified as bacteria that produce indole from Trp [77]. The spectrum of microbial indole derivatives, metabolic pathways utilized in the synthesis of these small compounds, and their biological activity in the host context, have shone more light on the physiological role of some members of human-associated microbiomes. In short, skatol (3-methylindole) produced by some strains of *Lactobacilli* and *Clostridia* genera, tryptamine synthetized by *Clostridium sporogenes* and *Ruminococcus gnavus,* and indolic acid derivatives, constitute the main bacterial Trp breakdown products [18]. Indole-3-acetic acid (IAA) produced by (among others) *Clostridia, Bifidobacter* and *Bacterioides* spp., indole pyruvic acid (IPA), a quite recently discovered metabolic by-product of *Clostridia* spp. *(Clostridia sporogenes),* and *Peptostreptococcus asscharolyticus* or indolealdehyde (IAld) synthetized by members of the *Lactobacillus* genus, are all indolic acid derivatives with known effects on the host immune system [18]. The enzyme tryptophanase (TnaA) is essential for converting Trp into indole and was considered an exclusive feature of the prokaryotic metabolome. However, recent reports have identified this bacterial-derived element, probably transmitted through lateral gene transfer [78], in the genome of *Blastocystis*, a eukaryotic member of the human gut-associated microbiome [79,80,81]. *Blastocystis* subtype 7, a human-derived subtype that is pathogenic in a mouse model and modulates microbiome composition (for example by reducing the presence of Bifidobacteria), expresses TnaA [82]. These data indicate that tryptophan metabolism transferred from the bacterial kingdom could provide an essential feature for adaptation of this eukaryotic parasite to the gut environment.

Besides being cross-metabolized by the different bacterial strains and regulating interspecies synergy within microbial consortia, Trp metabolites, particularly indolic acid derivatives, constitute an important element of the host–microbiome interactome. The main pathways targeted by these indole derivatives in the host compartment are those associated with activation of the transcription factors aryl hydrocarbon receptor (AhR) and pregnane X receptor (PXR) [83,84]. AhR is an intracellular environmental sensor activated by xenobiotic (2,3,7,8-tetrachlorodibenzo-*p*-dioxin (TCDD)), endogenous (6-formylindolo [3,2-b]carbazole (FICZ)), and microbial-derived (tryptophan metabolites like indolic acid derivatives, IAA, IAld) agonists [18,85,86]. Activation by agonistic ligands results in dissociation of the heat shock protein 90-α (HSP90) chaperone from the inactive AhR complex and translocation of AhR into the nucleus. In the nucleus, AhR binds specific sites in genomic DNA, and as such, controls the transcription of defined genes and activates several different signaling pathways. Hence, triggering the cascades downstream from AhR occurs in both xenobiotic response element (XREs)-dependent and XRE-independent manners. Additionally, AhR can also interact by directly signaling through the pro-inflammatory nuclear factor-κB (NF-κB) signaling pathway [87]. The cytochrome P450 enzymes Cyp1a1, 1a2, and 1b1 are upregulated upon AhR activation by agonist ligands. Interestingly, in a mouse sepsis model, Cyp1a1 was recently described as a possible intrinsic proinflammatory factor, acting by orchestrating macrophages to augment IL6 production, a cytokine known to have a negative impact on Foxp3 expression [88,89].

PXR is another receptor that has been recently deorphanized in the context of microbial-produced indole derivatives [83]. From the broad range of Trp metabolites, IPA constitutes a well-defined ligand for PXR [83,90]. It is worth noting that this particular compound was originally considered as an AhR ligand, however in the experimental conditions utilized by Venkatesh et al., IPA had no visible effects on this receptor.

### 4.2. Microbial-Derived Indole Derivatives and Control of Adaptive Immunity

Upregulation of PXR upon T cell stimulation, as well as higher responsiveness of PXR-deficient T lymphocytes, indicates the role of this receptor during an antigen-driven response [91]. At the same time, IPA was recently shown to be a microbial-derived PXR ligand [83]. Experiments done on mouse models colonized with *Clostridium sporogenes,* to date, the specie considered to be the sole source of IPA in the gut, and an IPA pathway-deficient *C. sporogenes* variant (fldC-deleted) revealed that deficiency of this metabolite caused CD4^+^ and CD8^+^ T cells to undergo increased expansion. Furthermore, responding T lymphocytes in the group colonized with the fldC mutant showed an elevated number of cells with antigen-experienced effector or memory phenotype (CD44^+^) [90]. These data might indicate a direct or indirect role of the IPA–PXR axis in the shaping of the T cell compartment, so the precise intrinsic outcome of the ligand–receptor interaction for individual T cell clones is still elusive.

Much more is known about the effect exerted by the microbial-derived Trp metabolites on T cell compartments through AhR signaling. The activation of AhR affects Th17 and Treg development. Interestingly, TCDD and FICZ are potent ligands for AhR, but drive opposite outcomes in regards to Th17 and Treg polarization [85,86,92]. Studies addressing these two compounds concentrated more on the biological and physical characteristics of the ligand–receptor interaction in relation to T cell lineage reprogramming, but it is intriguing that TCDD is a xenobiotic and FICZ an endogenous AhR ligand. Thus, these findings provide insight into possible roles of AhR ligands produced by gut-dwelling consortia in shaping of T cell differentiation. Furthermore, indole-3-acetaldehyde (I3AA), an indole derivative produced by intestinal microbiota, can serve as a substrate for the light-independent endogenous synthesis of FICZ, indicating a possible interplay between pathways related to environmental and endogenous ligands [93]. TCDD induces Treg, while FICZ induces Th17 cell generation in vitro [87,94,95]. Importantly, these potential anti- and pro-inflammatory characteristics associated with Treg and Th17 induction by TCDD and FICZ, respectively, were mirrored in experimental autoimmune encephalomyelitis (EAE) and IBD models [95,96]. This AhR-associated dualism is explained by the duration of the receptor–ligand interaction that depends in part on differential susceptibility to the intracellular breakdown of the AhR ligands by Cyp1a1 [97,98]. Indeed, AhR plays a pivotal role in the generation of fully functional Th17 T cells. This particular T cell subset is characterized by high AhR expression. Although AhR-deficient T cells undergo Th17 differentiation in response to transforming growth factor beta TGFβ and IL6, the polarized cells lack the expression of IL22 [94]. This phenomenon is explained by impaired recruitment of retineic-acid-receptor-related orphan nuclear receptor gamma RORγt to the IL22 promoter upon AhR deficiency. Additionally, AhR acts in a negative loop with signal transducer and activator of transcription (STAT)1 and STAT5, negative regulators of Th17 differentiation [99]. Under particular circumstances, agonistic AhR stimulation by hypoxia-inducible factor 1-α (HIF1α) degradation favors the expression of anti-inflammatory IL10 and promotes conversion towards Tr1 cells (Foxp3^−^, IL10^+^ suppressor T cells) [100]. Additionally, AhR plays a crucial role in the modulation of Foxp3 and has been linked to the T-cell immunoreceptor with Ig and ITIM domains (TIGIT)-expressing Tregs, a unique subset involved in Th1 and Th17 inhibition [101]. Finally, Treg generation can be promoted in vivo by specialized antigen presenting cells. Most reports indicate anti-inflammatory actions of AhR activation in regard to DC and Treg polarization [102,103] Hence, whether AhR stimuli will promote pro- or anti-inflammatory responses likely depends on the type of ligands and to the broader context of activation linked to cytokine expression and other factors within the milieu.

Another group of T cells which rely on AhR stimuli are the TCRαβ and TCRγδ intraepithelial lymphocytes [104]. It has been demonstrated that signaling through AhR is crucial for their functional maintenance, and for survival of TCRαβ CD8αα IEL in the mouse gut. In an experimental murine model, AhR ligands produced by *Lactobacillus reuteri* induced the generation of anti-inflammatory TCRαβ CD4^+^ CD8αα IEL (double positive (DP) IEL [105]. The conversion of single positive (SP) CD4^+^ IEL towards the DP phenotype was related to the downregulation of the T helper-inducing POZ (poxvirus and zinc finger)/Krüppel-like factor (ThPOK) in response to AhR stimulation.

A potential role for AhR in immune tolerance and protection has been demonstrated broadly in animal models [94,106]. Malfunction of AhR signaling and Trp metabolism within the microbiome metabolome was recently linked to human inflammatory bowel disease (IBD), an autoimmune disorder related to an aberrant immune response associated with the T cell compartment [107,108,109,110]. Decreased AhR expression and thus impaired AhR signaling was observed in gut tissue from IBD patients [111]. Less diversity was found in bacterial consortia from IBD-derived fecal samples [112,113]. The observed dysbiosis might corelate with the dysregulation of Trp metabolism within the microbial metabolome [114,115]. Indole acrylic acid, a Trp metabolite produced by *Peptostreptococcus* spp., inhibits secretion of the pro-inflamatory cytokines IL1β, IL6, and tumor necrosis factor (TNF) in human peripheral blood mononuclear cells and ameliorates colitis in a mouse model [114,115]. Patients who suffer graft versus host disease (GvHD) after allogeneic hematopoietic stem cell transplantation exhibit a shift in the microbial metabolome [116]. As pointed out by others, this negatively affects the production of indole compounds from tryptophan metabolism [117]. Interestingly, two different groups addressing GvHD in mouse models revealed possible protective [118] or detrimental [119] roles of T cell-related AhR signaling during development of pathological symptoms.

### 4.3. Bile Acid Metabolism 

Bile acids constitute products of co-metabolism by both host and microbiome. A broad array of these compounds are engaged in promoting nutrient absorption and regulating nutrient metabolism by acting as hormones [120]. Secondary bile acids can act in multiple ways as signaling molecules, through activation of the nuclear Farnesoid X receptor (FXR), vitamin D receptor, and through surface expressed GPCRs. The spectrum and distribution of gut bile acids and their derivatives depends, to a large extent, on the composition of the microbiome [121]. Engagement of these compounds in many physiological aspects has been appreciated for some time, but a role for bile acids as controllers of the Treg–Th17 axis has emerged only recently. Two such compounds, isoallo lithocholic acid (LCA) and 3-oxoLCA, were identified from a library of 30 compounds [122]. induces Treg development, while 3-oxoLCA inhibits Th7 differentiation. This effect was interpreted as being due to modulation of the RORγt transcription factor. Another bile acid metabolite, 3β-hydroxydeoxycholic acid (isoDCA), has a positive effect on the Treg compartment [123]. The proposed mechanism for the promotion of peripheral Treg generation was Farnesoid X receptor activation in DCs, leading to impaired immunostimulatory properties of these cells. All these data collectively indicate that bile acid metabolism is an important player in gut homeostasis. This notion is strongly supported by fecal metabolome data from IBD cohorts, where changes in gut consortia composition with evident dysregulation of bile acid metabolism was reported [115,124].

### 4.4. Vitamin B2 Metabolites

Mucosal associated invariant T (MAIT) cells constitute an innate-like T cell compartment characterized by unique TCR features. Biases of the MAIT cell repertoires are reflected by selective use of the T cell receptor α variable gene TRAV1-2 with T cell receptor α joining segments TRAJ33, TRAJ12, or TRAJ20 in the TCRα chain, and T cell receptor β variable genes TRBV6 or TRBV20 in the TCRβ chain [125,126]. Hence, the TCR repertoire diversity in this highly invariant compartment is restricted to the complementarity-determining region (CDR) 3. In contrast to conventional TCRαβ T cells that are engaged in recognition of the peptide moiety presented on MHC class I or II, MAITs are activated by bacterial metabolites presented in the context of the non-polymorphic MHC class I-related molecule, MR1 (Major histocompatibility complex class I-related protein 1) [127]. The identified activators of MAITs belong to a group of compounds related to vitamin B2 (riboflavin) metabolism, for instance the bacterial product 5-(2-oxopropylideneamino)-6-D-ribitylaminouracil (5-OP-RU) [127,128]. The riboflavin biosynthesis pathway constitutes a determining feature of the immune response associated with MAIT cell activation, attributed to specific bacterial species. Hence, MAITs provide protection against pathological species like *S. pneumoniae, Klebsiella pneumoniae, Salmonella enterica* serovar Typhimurium, *Escherichia coli, Pseudomonas aeruginosa*, and *Mycobacterium tuberculosis* [129,130]. Interestingly, the canonical development model of MAITs divides this process into two stages. The first is central programming in the thymus, where the commitment of CD4^+^8^+^ DP thymocytes to the MAIT lineage occurs in the absence of cognate, bacterial-derived ligands [131]. A second, peripheral phase with final polarization, occurs upon encounter with bacterial metabolites [130]. However, a recent report reveals that bacteria-derived 5-OP-RU may also play an essential role in the thymic phase of MAIT development [128]. This indicates that the microbiome’s role regarding MAIT-associated immunity is not restricted to the local presentation of metabolites in mucosal tissue. The composition of the gut consortia has a profound impact on this invariant T cell subset in the initial stages of development.

## 5. Conclusions

The adaptive immune compartment constitutes a crucial element that contributes to the maintenance of overall physiological homeostasis. The functional profile of the T cell subsets and their peripheral plasticity contributes to the complexity of this regulation. Crosstalk between many different molecular and cellular elements defines the pro- or anti-inflammatory outcome. Maintenance of the balance between effector and regulatory components requires sophisticated mechanisms to control the courses of action of functionally different clones in the steady state and during pathological events. To cope with this task, the immune system relies on intrinsic and exogenous stimuli that, to a large extent, are due to the microbiome with its broad spectrum of metabolites. In this review, we have summarized the cellular mechanisms related to some microbiota-derived metabolites, that through T cell-mediated processes, contribute to the development or regression of pathological states. The majority of data revealing the impact of individual microbial-produced metabolites on the immune compartment originate from mouse models. Thus, the real connections between the metabolome of the gut-dwelling microbial consortia and human health remain somewhat uncertain.

## Figures and Tables

**Table 1:** Main SCFAs, preferentially produced by some members of gut residing bacterial species.

SCFA	Species	Ref
Butyrate	*Roseburia intestinalis*	[26]
	*Eubacterium rectale*	
	*Eubacterium hallii*	
	*Ruminococcus obeum*	
	*Ruminococcus gnavus*	
Propionate	*Clostridium ramosum*	[27]
	*Clostridium bifermentans*	
	*Bacteroides fragilis*	
Acetate	*Clostridium ramosum*	[26,27]
	*Clostridium bifermentans*	
	*Bacteroides fragilis*	
	*Ruminococcus obeum*	
	*Ruminococcus gnavus*	
Valerate/Pentanoate	*Clostridium luticellarii*	[28]
	*Candidatus methanogranum*	
